# Supplementary material for: Impact of Camellia japonica Bee Pollen Polyphenols on Hyperuricemia and Gut Microbiota in Potassium Oxonate-Induced Mice
Source: Nutrients. 2021 Jul 30;13(8):2665. doi: 10.3390/nu13082665 (PMC8401623; doi:10.3390/nu13082665)
Supplement: Supplementary file 1 [file nutrients-13-02665-s001.zip › nutrients-1303592-Supplementary Materials.pdf]

Table S1 Primer sequences

Gene	Forward primer	Reverse primer	bp
β-Actin	GTGACGTTGACATCCGTAAAGA	GTAACAGTCCGCCTAGAAGCAC	287
URAT1	GTGACGTTGACATCCGTAAAGA	GTAACAGTCCGCCTAGAAGCAC	93
GLUT9	GAGATGCTCATTGTGGGACG	TCACTCCGAACAGGTATGGC	220
OAT1	CACAATGATTCGGCAGACGG	AGGTATGGAGGGGTAGAACTCG	109
OCT1	ACATCCATGTTGCTCTTTCG	TTGCTCCATTATCCTTACCG	315
ABCG2	ATGTTGTGATGGGCACTCTGAC	TATCCACACAGGGAAAGTCCTACT	210
TLR4	CCTGGACTTGGACCTCAG	GGACTGAAAGCTGCACATC	156
MYD88	TCGCGCATCGGACAAACG	GCAATGGACCAGACACAGGT	288
NF-κB	AGTTGAGGGGACTTTCCCAGGC	GATTCGAGTATTAGTTCATGGA	132
NLRP3	AAGGAAGTGGACTGCGAGAA	ACGTTCGTCCTTCCTTCCTT	110
ASC	GTGGGTGGCTTTCCTTGATT	TTGTCTTGGCTGGTGGTCTCT	229
Caspase-1	AACTGGAGCTGAGGTTGACA	TCAGAGGTCTTGTGCTC TGG	147

Table S2 Effect of CPE-E on the relative abundance (%) at the phylum level of gut microbiota

Phylum-level Microorganisms	Group I	Group II	Group III	Group IV	Group V
Firmicutes	51.25 ± 13.47^{b}	78.98 ± 19.54^{a}	64.06 ± 15.33^{ab}	52.08 ± 14.35^{b}	63.62 ± 17.68^{ab}
Bacteroidetes	31.20 ± 8.57^{a}	14.75 ± 4.35^{b}	28.74 ± 6.12^{a}	32.10 ± 7.43^{a}	17.68 ± 4.67^{b}
Actinobacteria	6.79 ± 2.21^{a}	2.52 ± 1.36^{b}	1.89 ± 1.74^{b}	8.67 ± 3.59^{a}	8.18 ± 3.42^{a}
Proteobacteria	7.27 ± 2.16^{a}	1.73 ± 1.02^{b}	2.93 ± 1.97^{b}	2.37 ± 1.71^{b}	5.96 ± 2.33^{a}
TM7	2.01 ± 0.92^{a}	0.85 ± 0.24^{b}	1.06 ± 1.34^{b}	2.16 ± 1.38^{a}	2.46 ± 1.09^{a}
Tenericutes	0.51 ± 0.33^{a}	0.48 ± 0.28^{a}	0.47 ± 0.34^{a}	0.50 ± 0.27^{a}	0.74 ± 0.53^{a}
Deferribacteres	0.39 ± 0.33^{b}	0.35 ± 0.31^{b}	0.25 ± 0.17^{b}	1.30 ± 0.85^{a}	0.57 ± 0.36^{b}
Cyanobacteria	0.21 ± 0.20^{a}	0.15 ± 0.13^{a}	0.24 ± 0.22^{a}	0.37 ± 0.33^{a}	0.43 ± 0.23^{a}
Nitrospirae	0.16 ± 0.15^{a}	0.00 ± 0.00^{a}	0.13 ± 0.08^{a}	0.22 ± 0.21^{a}	0.12 ± 0.11^{a}
[Thermi]	0.00 ± 0.00^{a}	0.00 ± 0.00^{a}	0.00 ± 0.00^{a}	0.10 ± 0.06^{a}	0.00 ± 0.00^{a}
others	0.21 ± 0.19^{a}	0.19 ± 0.06^{a}	0.23 ± 0.09^{a}	0.13 ± 0.05^{a}	0.24 ± 0.13^{a}

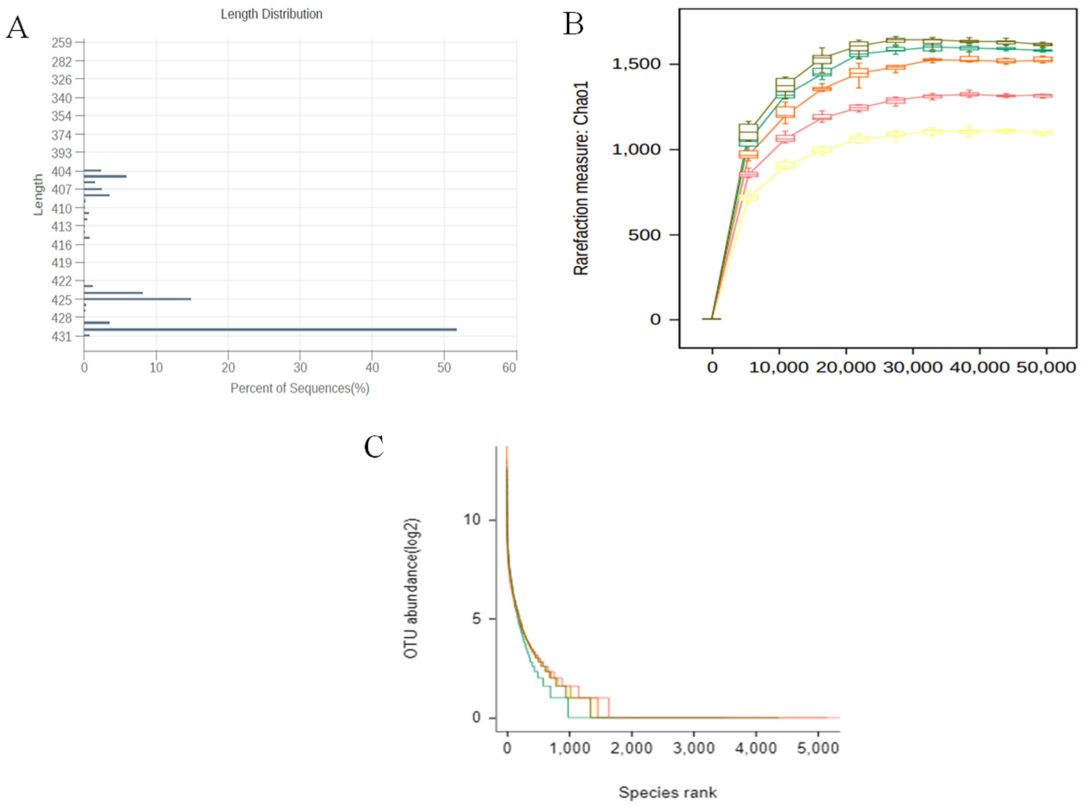

Figure S1 DNA sequencing number and length (A), rarefaction curve (B), rank abundance curve (C)
